# Susceptibility to glaucoma: differential comparison of the astrocyte transcriptome from glaucomatous African American and Caucasian American donors

**DOI:** 10.1186/gb-2008-9-7-r111

**Published:** 2008-07-09

**Authors:** Thomas J Lukas, Haixi Miao, Lin Chen, Sean M Riordan, Wenjun Li, Andrea M Crabb, Alexandria Wise, Pan Du, Simon M Lin, M Rosario Hernandez

**Affiliations:** 1 Department of Molecular Pharmacology and Biological Chemistry, Feinberg School of Medicine, Northwestern University, E Chicago Ave, Chicago, IL 60611 USA; 2 Department of Ophthalmology, Feinberg School of Medicine, Northwestern University, E Chicago Ave, Chicago, IL 60611 USA; 3 Department of Biology, City College of New York, Convent Ave, New York, NY 10031, USA; 4 Robert H Lurie Comprehensive Cancer Center, Feinberg School of Medicine, Northwestern University, E Chicago Ave, Chicago, IL 60611 USA

## Abstract

**Background:**

Epidemiological and genetic studies indicate that ethnic/genetic background plays an important role in susceptibility to primary open angle glaucoma (POAG). POAG is more prevalent among the African-descent population compared to the Caucasian population. Damage in POAG occurs at the level of the optic nerve head (ONH) and is mediated by astrocytes. Here we investigated differences in gene expression in primary cultures of ONH astrocytes obtained from age-matched normal and glaucomatous donors of Caucasian American (CA) and African American (AA) populations using oligonucleotide microarrays.

**Results:**

Gene expression data were obtained from cultured astrocytes representing 12 normal CA and 12 normal AA eyes, 6 AA eyes with POAG and 8 CA eyes with POAG. Data were normalized and significant differential gene expression levels detected by using empirical Bayesian shrinkage moderated t-statistics. Gene Ontology analysis and networks of interacting proteins were constructed using the BioGRID database. Network maps included regulation of myosin, actin, and protein trafficking. Real-time RT-PCR, western blots, ELISA, and functional assays validated genes in the networks.

**Conclusion:**

Cultured AA and CA glaucomatous astrocytes retain differential expression of genes that promote cell motility and migration, regulate cell adhesion, and are associated with structural tissue changes that collectively contribute to neural degeneration. Key upregulated genes include those encoding myosin light chain kinase (*MYLK*), transforming growth factor-β receptor 2 (*TGFBR2*), rho-family GTPase-2 (*RAC2*), and versican (*VCAN*). These genes along with other differentially expressed components of integrated networks may reflect functional susceptibility to chronic elevated intraocular pressure that is enhanced in the optic nerve head of African Americans.

## Abstract 2

Comparison of gene expression in normal and glaucomatous eyes from Caucasian American and African American donors reveals differences that might reflect different susceptibility to glaucoma.

## Background

Glaucoma comprises a group of diseases that are characterized by optic neuropathy associated with optic disc cupping and loss of visual field and, in many patients, with elevated intraocular pressure (IOP) [1]. There are several types of glaucoma, including juvenile and adult-onset types, primary open angle glaucoma (POAG), narrow-angle glaucoma, and secondary glaucoma, with different pathogenic mechanisms. POAG is more prevalent in Black Americans of African American (AA) ancestry than in Caucasian American (CA) populations of European ancestry (CA), with reported frequencies of 3-4% in the AA population over the age of 40 years, as compared with approximately 1% in CA populations [2]. The disease is particularly frequent in Afro-Caribbean persons, with a prevalence of 7% in Barbados and 8.8% in St Lucia [3]. On average, African Americans have the longest duration [4] and higher progression of disease [5] compared to other populations. In addition to racial differences, a positive family history of POAG is a major risk factor for the disease in African Americans [6]. The Advanced Glaucoma Intervention Study (AGIS), which compared the glaucoma outcomes in AA and CA patients, concluded that after failure of medical therapy, surgical trabeculectomy delayed progression of glaucoma more effectively in CA than in AA patients [7,8].

Abnormally elevated IOP elicits a complex sequence of putative neurodestructive and neuroprotective cellular responses in the optic nerve head (ONH) [9]. Previous studies demonstrated that gene expression in astrocytes of the glaucomatous ONH serve as the basis for these responses [10]. Here we present evidence that primary cultures of AA and CA astrocytes derived from POAG donors exhibit differential gene expression of genes that relate to reactive astrocytes and to pathological changes that occur in the glaucomatous ONH. Validations of changes in expression of selected genes were done by quantitative real-time RT-PCR, western blots, enzyme-linked immunosorbent assay (ELISA) and various functional assays. Network analysis of gene product interactions focused our findings on specific functional pathways. Our data indicate that both normal and glaucomatous astrocytes from AA donors exhibit differential expression in genes that regulate signal transduction, cell migration, intracellular trafficking and secretory pathways.

## Results and discussion

### Primary cultures of ONH astrocytes from normal and glaucomatous donors

#### Demographics and clinical history

Demographic characteristics of the normal AA and CA donors used in this study are detailed in Additional data file 2. Demographic and clinical data for AA donors with glaucoma (AAGs) and CA donors with glaucoma (CAGs) included in the microarray analyses and other assays are detailed in Additional data file 1. Twelve eyes from ten CAG donors and six eyes from AAG donors were used in this study. Glaucoma drug treatment history was available for some POAG donors. None of the drug treatments are known to affect astrocytes in the ONH. The degree of glaucomatous damage in donors with POAG was assessed using histories when available and by evaluating axon degeneration in cross-sections of the myelinated optic nerve (Additional data file 1). A limitation of this study is that only six eyes from three AAG donors were available due to the extreme rarity of these samples. Consequently, we used all six eyes to generate primary cultures for all experiments in our study. Primary cultures of samples from AAG and CAG donors were fully characterized as ONH astrocytes as described in detail earlier [11].

### Identification of differentially expressed genes in ONH astrocytes from AA and CA donors with POAG

#### Comparisons

For the comparisons amongst the four groups, our primary focus was to establish the differentially expressed genes between AAG and CAG donors (Additional data file 7); our secondary focus was the comparison between normal and glaucomatous astrocytes and our tertiary focus was to identify differentially expressed genes within each population: AAG versus AA and CAG versus CA.

The comparisons allowed us to identify the unique gene expression profile in AAG astrocytes compared to CAG astrocytes and AAG compared to AA (Additional data file 8). In addition, we identified a common group of genes that exhibit a similar gene expression pattern in both AAG and CAG compared to normal AA and CA astrocytes, which we named common glaucoma-related genes (Tables 1 and 2).

**Table 1:** Common genes significantly decreased in glaucomatous ONH astrocytes compared to their normal counterparts

			AAG-AA (U133Av2)		CAG-CA (U95Av2)	
				
Symbol	Description	CL	FC	*p*-value	FC	*p*-value
*AMIGO2*	Adhesion molecule with Ig-like domain 2	12q13.11	-1.52	0.0498	-2.01	0.0011
*BMP1*	Bone morphogenetic protein 1	8p21	-1.92	0.0005	-2.08	0.0001
*CD97*	CD97 molecule	19p13	-1.65	0.0015	-1.36	0.0008
*CRIP2*	Cysteine-rich protein 2	14q32.3	-2.58	0	-1.44	0.0034
*DGKA*	Diacylglycerol kinase, alpha 80 kDa	12q13.3	-1.54	0.0034	-1.28	0.0001
*DMPK*	Dystrophia myotonica-protein kinase	19q13.3	-2.45	0	-1.62	0.0021
*EFHD1*	EF-hand domain family, member D1	2q37.1	-4	0	-2.01	0.0011
*GPC1*	glypican 1	2q35-q37	-1.61	0.0032	-1.31	0.0026
*MGLL*	Monoglyceride lipase	3q21.3	-1.52	0.0083	-1.75	0.0005
*MICAL2*	Microtubule associated monoxygenase, calponin and LIM domain containing 2	11p15.3	-1.62	0.0186	-2.02	0.0013
*NPAL3*	NIPA-like domain containing 3	1p36.12-p35.1	-1.54	0.0034	-1.51	0.0079
*PDGFA*	Platelet-derived growth factor alpha polypeptide	7p22	-1.65	0.0076	-2.21	0.0004
*SLC12A2*	Solute carrier family 12, member 2	5q23.3	-1.61	0.0032	-1.51	0.0001
*SLC12A4*	Solute carrier family 12, member 4	16q22.1	-2.42	0.0007	-1.19	0.0046
*SMTN*	Smoothelin	22q12.2	-1.79	0.0162	-1.99	0.001
*WWP2*	WW domain containing E3 ubiquitin protein ligase 2	16q22.1	-1.87	0.0006	-1.39	0.0029

CL, chromosome location; FC, fold change.

**Table 2:** Differentially expressed genes in glaucomatous astrocytes*

Gene	Description	FC	*p*-value	CL
**Genes associated with myosin regulation**				
*CALM1*	Calmodulin 1	2.23^†^	0.00121	14q24-q31
*MYH10*	Myosin, heavy chain 2	1.64	0.00588	17p13.1
*MYLK*	Myosin, light polypeptide kinase	2.89	0.000133	3q21
*PIK3R1*	Phosphoinositide-3-kinase, subunit (p85-alpha)	1.62	0.00201	5q13.1
*MYPT1*	Protein phosphatase 1, regulator subunit 12A (PPP1R12A)	1.51	0.000775	12q15-q21
*RAC2*	Ras-related 2 (Rho family, Rac2)	2.34	0.001059	22q13.1
*RPS6KA3*	Ribosomal protein S6 kinase, 90 kDa, polypeptide 3	1.5	0.000061	Xp22.2-p22.1
				
**Genes associated with actin regulation**				
*ARHGEF7*	Rho guanine nucleotide exchange factor (GEF) 7	1.71	0.000064	13q34
*NCK1*	NCK adaptor protein 1	1.64^†^	0.000015	3q21
*PDLIM1*	PDZ and LIM domain 1 (elfin, CLP36)	1.61	0.00106	10q22-q26.3
*PIK3R1*	Phosphoinositide-3-kinase, regulatory subunit 1	1.61	0.002012	5q13.1
*PLEC1*	Plectin 1, intermediate filament binding protein	-1.82	0.00199	8q24
*PTPN11*	Protein tyrosine phosphatase, non-receptor type 11	-1.9	0.000005	12q24
*RAC2*	Ras-related 2 (Rho family, Rac2)	2.34	0.001059	22q13.1
*SMAD3*	SMAD, mothers against DPP homolog 3	1.9	0.000488	15q22.33
*TGFBR1*	Transforming growth factor, beta receptor I	-1.57	0.000038	9q22
*TGFBR2*	Transforming growth factor, beta receptor II	2.11	0.007253	3p22
				
**Genes associated with protein trafficking**				
*APPBP1*	Amyloid beta precursor protein binding protein 1	1.62	0.001688	16q22
*CCL5*	Chemokine (C-C motif) ligand 5	-1.74	0.002283	17q11.2-q12
*CDH2*	Cadherin 2, type 1, N-cadherin (neuronal)	1.55	0.003173	18q11.2
*COL4A4*	Collagen, type IV, alpha 4	1.59	0.002335	2q35-q37
*CTNNB1*	Catenin (cadherin-associated protein), beta 1, 88 kDa	2.14	0.005445	3p21
*CTNND1*	Catenin (cadherin-associated protein), delta 1	1.68	0.000025	11q11
*GOLGA1*	Golgi autoantigen, golgin subfamily a, 1	1.51	0.00002	9q33.3
*GOLGA2*	Golgi autoantigen, golgin subfamily a, 2	1.77	0.000002	9q34.11
*GOLGA3*	Golgi autoantigen, golgin subfamily a, 3	1.97	0.000128	12q24.33
*HAPLN1*	Hyaluronan and proteoglycan link protein 1	8.04	0.001193	5q14.3
*PRSS3*	Protease, serine, 3 (mesotrypsin)	2.53	0.005135	9p11.2
*RAB1A*	RAB1A, member RAS oncogene family	1.51	0.000274	2p14
*RAB4A*	RAB4A, member RAS oncogene family	1.52	0.00035	1q42-q43
*RAB5B*	RAB5B, member RAS oncogene family	1.5^‡^	0.0081	12q13
*RAB9A*	RAB9A, member RAS oncogene family	1.64	0.000256	Xp22.2
*RAB9P40*	RAB9 effector protein with kelch motifs	1.84	0.000002	9q33.3
*RABGGTB*	Rab geranylgeranyltransferase, beta subunit	1.76	0.000375	1p31
*TGM2*	Transglutaminase 2	2.75	0.008289	20q12
*VCAN*	Versican (chondroitin sulfate proteoglycan 2, CSPG2)	2.94	0.000265	5q14.3

*Genes differentially expressed in AAG compared to CAG (Additional data file 7) except where noted. ^†^From Additional data file 5. ^‡^From qRT-PCR data (Figure 3b). FC, fold change; CL, chromosome location.

Eight eyes from six CAG donors were used to generate astrocytes for eight Hu95v2 chips. Six eyes from three AAG were used to generate astrocytes for six Hu95Av2 chips and six Hu133A 2.0 chips. Eighteen Hu133 2.0 chips from nine normal AA and nine normal CA donors, and seven Hu95v2 chips from six normal CA donors were used for comparisons within the appropriate platform. All microarray data have been deposited in the NCBI GEO database under the series accession number GSE9963.

The data measured by the two types of chips were normalized separately by RMA normalization as described in Materials and methods. Differentially expressed genes required an up or down fold-change of more than 1.5-fold (*p *< 0.01, false discovery rate < 0.05). A total of 618 genes were differentially expressed in AAG-CAG comparisons, 484 upregulated and 134 downregulated (Additional data file 7); 509 genes were differentially expressed in AAG compared to normal AA astrocytes, 167 upregulated and 342 downregulated (Additional data file 5); and 195 genes were differentially expressed in the CAG-CA comparison, 132 upregulated and 63 downregulated (Additional data file 6). We used empirical Bayesian methods to identify differentially expressed genes; both our results (not shown) and previous studies [12,13] have suggested that the empirical Bayesian method has performance similar to statistical analysis of microarrays (SAM). To reduce batch effects, we added fold-change criteria because genes with larger fold-change are less likely to be affected by such effects.

#### Gene Ontology

Gene Ontology (GO) analysis of differential expression in glaucomatous astrocytes was done with GoMiner [14]. There were 33 significant categories for CAG-CA, 80 for AAG-AA, and 67 for AAG-CAG comparisons (*p *< 0.01). The significant genes in selected categories were mined using GOstats in Bioconductor (Additional data file 9). The phosphorylation category (GoID: 16310) was significant in the three datasets. The percent distribution of the genes common to all of the datasets in this category was determined (Additional data file 10). For example, the genes encoding myosin light chain kinase (*MYLK*) and calcium/calmodulin-dependent serine protein kinase (*CASK1*) were found in all three glaucoma comparisons. Those encoding the regulatory subunit of phosphatidylinositol-3-kinase (*PIK3R1*), transforming growth factor (TGF)β-receptor 2 (*TGFBR2*), ERBB2, and Ephrin receptor A5 were some of the genes found in two datasets (AAG-CAG and AAG-AA). Similarly, another category with overlaps between the datasets was cell-cell signaling (Additional data file 10). Some of the genes in this category include those encoding latent transforming growth factor beta binding protein 4 (*LTBP4*), the glutamate receptor subunit (*GRIK2*), and parathyroid hormone-like protein (*PTHLH*). As we show below, expansion of these and other GO categories using network-protein interaction software yielded three networks that include differentially expressed GTPases, protein kinases, transmembrane receptors, and proteins involved in trafficking at cellular membranes. Altogether, the GO analysis suggests that alterations in the signaling networks that regulate cell motility, polarity, adhesion, and trafficking are present in glaucomatous astrocytes. Moreover, the overlap among the datasets in multiple categories suggests that there is a spectrum of changes in gene expression in glaucoma.

#### Network analysis

**Figure 1:**
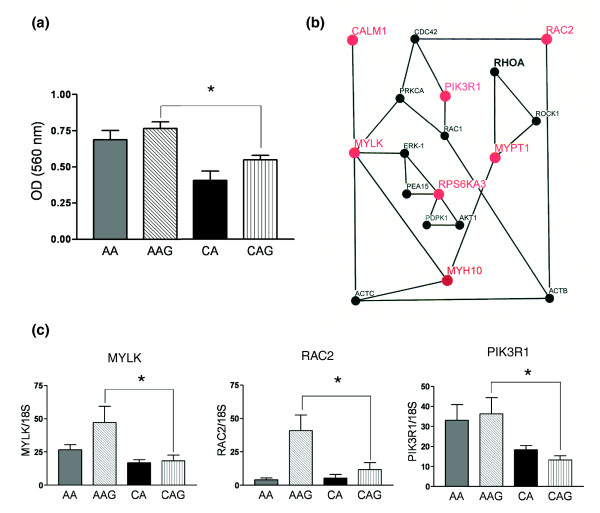
Astrocyte migration and the myosin regulatory network in glaucoma astrocytes. **(a) **Cell migration assay shows that AA and AAG astrocytes migrate significantly faster than CA and CAG astrocytes. The assay was performed as described in the Materials and methods. Values represent mean optical density (OD) ± standard deviation of triplicate experiments using primary astrocyte cultures of six AA, five AAG, five CA and five CAG donors. Asterisk indicates *p*-value < 0.05. **(b) **Schematic representation of the myosin regulatory network. Upregulated mRNAs have large red nodes and font while downregulated mRNAs have large blue nodes and font. Small black nodes and font show genes have 'present calls' without differential expression. **(c) **Confirmation of three differentially expressed genes from myosin network by qRT-PCR in human ONH astrocytes: MYLK, RAC2 and PIK3R1. Genes were normalized to 18S RNA. Graphical representation of the relative mRNA levels in normal and glaucomatous AA and normal and glaucomatous CA astrocytes (n = 6, two-tailed *t*-test). Asterisk indicates *p *< 0.05).

**Figure 2:**
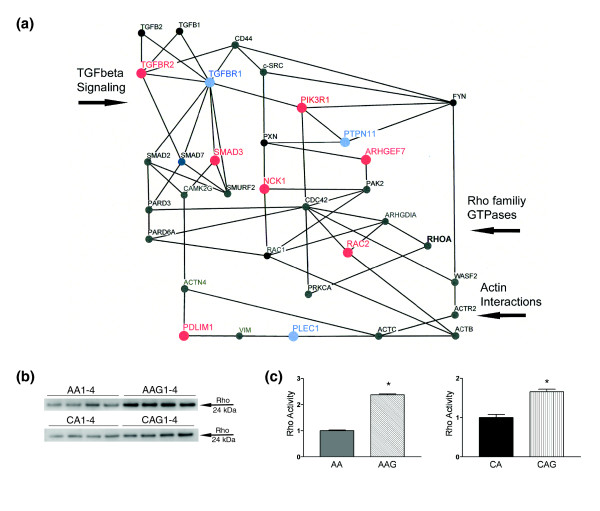
Actin regulatory network and TGFβ signaling in AAG astrocytes. **(a) **Schematic representation of the actin and TGFβ regulatory network. Upregulated mRNAs have large red nodes and capital font, while downregulated mRNAs are shown with large blue nodes and capital font. Small black nodes and capital font indicate genes that have 'present calls' without differential expression. The RhoA GTPase is in bold in black because of higher activity in glaucoma astrocytes. **(b) **Representative western blot of the pull-down Rho activation assay demonstrated that both AAG and CAG astrocytes exhibit significantly higher Rho activity than normal astrocytes under unstimulated conditions. **(c) **Densitometry analysis of the blots from Rho activation assay. Bars show mean fold difference in density ± standard error of two independent experiments. (Asterisk indicates *p *< 0.05)

**Figure 3:**
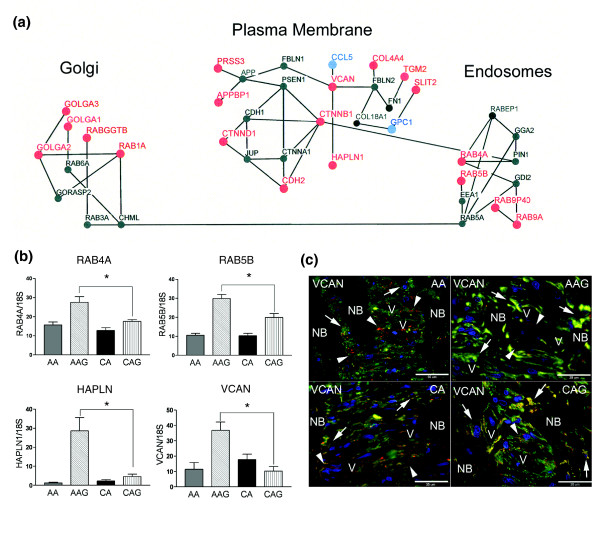
Intracellular trafficking networks associated with golgi, plasma membrane, and endosomes that have differentially expressed genes in glaucoma astrocytes. **(a) **Schematic representation of the intracellular trafficking network. Upregulated mRNAs have a large red node and font, while downregulated genes have a large blue node and font. Small black nodes and font indicate genes that have 'present calls' without differential expression. **(b) **Confirmation of four differentially expressed genes from the trafficking network by qRT-PCR in human ONH astrocytes: *RAB4A*, *RAB5B*, *HAPLN *and *VCAN*. Genes were normalized to 18S RNA. Graphical representation of the relative mRNA levels in normal and glaucomatous AA and normal and glaucomatous CA astrocytes (n = 6, two-tailed *t*-test). Asterisk indicates *p *< 0.05. **(c) **Representative double immunofluorescent staining of versican (VCAN; red) and astrocyte marker GFAP (green) in sections of human ONH from an AA donor (51 year old female), AAG donor (70 year old male), CA donor (70 year old male) and CAG donor (76 year old male). Nuclei (blue) are stained with DAPI. Note staining of VCAN (red) in the cribriform plates and surrounding the blood vessels (arrowheads). Arrows indicate versican co-localized with GFAP in astrocytes in the cribriform plates of the lamina cribrosa. VCAN staining is stronger in astrocytes of the glaucomatous lamina cribrosa. V, blood vessel; NB, nerve bundle. Scale bar 35 μm.

Three detailed network maps were constructed from the differential gene expression data. We focused mainly on the differences between AAG and CAG as this difference represents the maximal differential expression group (Additional data file 7). The networks include regulation of myosin, actin, TGFβ signaling and protein trafficking. For the myosin network, the initial node was myosin light chain kinase (MYLK) (Figure 1b). The actin regulatory networks were initiated using the TGFβ receptors (Figure 2a), and the protein trafficking networks were initiated using GOLGA3, catenin beta1 (CTNNB1) and RAB4A as nodes (Figure 3a). These were expanded using the BioGrid database for protein-protein interactions. In each network graph, the differentially expressed genes are shown by large nodes and font (red for increased, blue for decreased expression), while the connecting genes that are not differentially expressed are shown by black smaller nodes and font. Expression data for network nodes that are differentially expressed in the AAG-CAG comparison (Additional data file 7) are included in Table 3. Some network nodes were also selected from differentially expressed genes in AAG-AA (Additional data file 5) and in common AAG-AA and CAG-CA comparisons (Tables 1 and 2). In the description of each network, we present selected experimental data that verify changes in gene expression and effects on function.

**Table 3:** Common genes significantly increased in glaucomatous ONH astrocytes compared to their normal counterparts

			AAG-AA (U133Av2)		CAG-CA (U95Av2)	
			
Symbol	Description	CL	FC	*p*-value	FC	*p*-value
*ABCA8*	ATP-binding cassette, sub-family A, member 8	17q24	2.34	0.0291	2.53	9.43E-05
*C5orf30*	Chromosome 5 open reading frame 30	5q21.1	1.57	0.0028	1.48	0.0042
*CASK*	Calcium/calmodulin-dependent serine protein kinase	Xp11.4	1.99	0.0064	1.31	0.002
*CASP4*	Caspase 4, apoptosis-related cysteine peptidase	11q22.2-q22.3	1.59	0.0007	1.9	0.0026
*GSTA4*	Glutathione S-transferase A4	6p12.1	1.25	0.005	1.85	5.21E-05
*GULP1*	GULP, engulfment adaptor PTB domain containing 1	2q32.3-q33	1.89	0.0023	1.38	0.0075
*HEPH*	Hephaestin	Xq11-q12	4.15	0.0021	1.88	0.0021
*HOXB2*	Homeobox B2	17q21-q22	1.59	0.0133	1.86	0.0014
*KCNK2*	Potassium channel, subfamily K, member 2	1q41	1.55	0.0489	1.52	0.0024
*KIAA1199*	KIAA1199	15q24	1.68	0.0152	1.94	0.0026
*LMO4*	LIM domain only 4	1p22.3	1.7	0.0034	1.83	0.0052
*MYH10*	Myosin, heavy polypeptide 10, non-muscle	17p13	1.64	0.0012	1.57	0.0017
*PYGL*	Phosphorylase, glycogen; liver	14q21-q22	1.47	0.0141	2.2	0.0025
*RBP1*	Retinol binding protein 1, cellular	3q23	2.2	0.0007	2.32	0.00073
*SERPING1*	Serpin peptidase inhibitor, clade G, member 1	11q12-q13.1	2.3	0.0064	1.86	0.0014
*SH3BP5*	SH3-domain binding protein 5	3p24.3	1.65	0.0407	2.74	4.87E-05
*SLIT2*	Slit homolog 2	4p15.2	1.6	0.0077	1.42	0.0027
*TINP1*	TGF beta-inducible nuclear protein 1	5q13.3	1.53	7.93E-05	1.36	0.0055

CL, chromosome location; FC, fold change.

### Cellular motility and migration in AAG astrocytes

Migration of reactive astrocytes is an important component in the remodeling of the ONH in glaucoma [15,16]. In glaucoma, reactive astrocytes migrate from the cribriform plates into the nerve bundles [9,17] and synthesize neurotoxic mediators such as nitric oxide and tumor necrosis factor (TNF)α, which may be released near the axons, causing neuronal damage [18,19]. Previous work in our laboratory demonstrated that human ONH astrocytes *in vitro *respond to elevated pressure predominantly with an increase in cell migration that may be relevant to axonal degeneration and tissue remodeling in glaucomatous optic neuropathy [20].

Here we provide *in vitro *data of differential astrocyte migration in astrocytes from AAG donors using a standardized migration assay. As shown in Figure 1a, migration of AAG astrocytes is significantly increased compared to CAG astrocytes and migration is faster in AA compared to CA astrocytes. Because multiple cellular processes impact cell motility and migration, we divided our analysis between two interacting networks that regulate myosin and actin.

#### Myosin-dependent astrocyte migration

From the microarray and quantitative RT-PCR (qRT-PCR) data, the following genes related to myosin regulation were differentially expressed in AAG: *MYLK*, *MYPT1*, *RAC2*, *CALM1*, *RPS6KA3*, *MYH10*, and *PIK3R1*. Shown in Figure 1b is the network of proteins associated with the phosphorylation of the regulatory light chain of myosin II and activation of myosin-ATPase (*MYH10*). Two network nodes are critical for the regulation of myosin. These include MYLK, a calmodulin-activated protein kinase that phosphorylates Ser19 on the myosin regulatory light chain and MYPT1, the regulatory subunit of myosin-light chain phosphatase, which dephosphorylates the myosin light chain. We found that both genes were expressed in AAG astrocytes at significantly higher levels than in CAG astrocytes (Table 3). Similarly, calmodulin (*CALM1*), the activator of MYLK is also upregulated in AAG astrocytes (Table 3)

The upregulation of *MYLK *suggests that the myosin regulatory system may exhibit increased responsiveness towards modulation by various cellular second messenger signaling systems such as Ca^2+^, diacylglycerol, and cyclic nucleotides [21]. Similarly, changes in expression of *RAC2 *indicate that other members of the Rho-family signaling network are altered in AAG astrocytes (Figure 1c). These changes allow us to predict that the myosin-regulated motility may be sensitized to signals from Ca2^+^, Rho GTPase, and growth/trophic factors coupled to the activation of phosphoinositides. Within the phosphoinositide pathway, *PIK3R1 *is upregulated in AAG astrocytes (Figure 1c). The PIK3R1 pathway is important for the motility of ONH astrocytes [22] and their responses to increased hydrostatic pressure [20]. PIK3R1 is the regulatory subunit of the lipid kinase that transforms phosphoinositide (4,5) biphosphate (PIP2) into the triphosphate (PIP3). PIP3 in turn mediates activation of several of the Rho GTPases as well as selected protein kinases. Thus, in AAG astrocytes, lipid-activated pathways that modulate astrocyte motility are altered.

ERK1 potentiates MYLK activity through phosphorylation [23] and interacts with PEA15 (Phosphoprotein enriched in astrocytes) [24]. The increased expression of the S6-family kinase (RPS6KA3) may compete with ERK1 for binding to the phosphoprotein PEA15 [25], potentially increasing the pool of active ERK1. Consistent with this finding, we have shown that ERK1 is activated in normal CA ONH astrocytes, under increased hydrostatic pressure and in experimental glaucoma in primates [26]. Thus, myosin-based motility may be influenced by changes in MYLK expression and potentiation through ERK1 activation under hydrostatic pressure.

**Figure 4:**
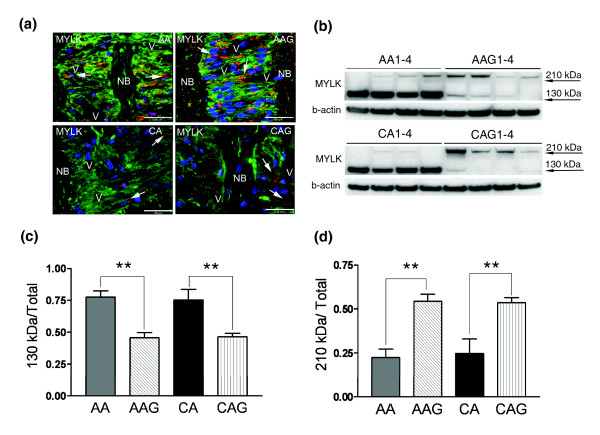
MYLK isoform expression in ONH astrocytes. **(a) **Representative double immunofluorescent staining of MYLK (red) and astrocyte marker GFAP (green) in sections of human ONH from an AA donor (51 year old male), AAG donor (70 year old male), CA donor (56 year old female) and CAG donor (76 year old male). Nuclei (blue) are stained with DAPI. Note strong granular staining of MYLK in astrocytes (arrows) in the cribriform plates of the lamina cribrosa of AA and AAG donors compared to CA and CAG donors. V, blood vessel; NB, nerve bundle. Scale bar 35 μm. **(b) **Representative western blots of astrocyte cell lysates with MYLK antibody. β-Actin was used as a loading control. Note that AAG1-4 donors express more MYLK-210 and less MYLK-130 than CAG1-4 donors. **(c) **Graph of MYLK-210 expressed as the fraction of MYLK-210 in the four groups. **(d) **Graph of the fraction of MYLK-130 expressed in the four groups. These results represent densitometry analysis of western blots using seven AA, five AAG, eight CA and eight CAG donor samples.

Co-localization of MYLK and glial acidic fibrillar protein (GFAP) by immunohistochemistry indicates that ONH astrocytes in tissue sections in the lamina cribrosa of normal AA and AAG expressed visibly higher levels of MYLK protein *in situ *(Figure 4a).

The *MYLK *gene has multiple genes within its locus [27]. In some tissues up to three transcripts are expressed, including for long and short forms of the kinase and a protein identical to the carboxyl-terminal sequence [27]. ONH astrocytes express both the 130 kDa (MYLK-130) and 210 kDa (MYLK-210) kinase isoforms and we quantified changes in both using standard densitometry measurements. Western blots (Figure 4b) show that the fraction of MYLK-210 in ONH astrocytes is higher in AAG and CAG compared to normal astrocytes, while the fraction of the MYLK-130 isoform decreases (Figure 4b). These differences were quantified using densitometry (Figure 4c, d). Thus, in glaucoma there appears to be MYLK isoform switching towards the larger protein. The difference between the two proteins is the presence of an amino-terminal extension in the 210 kDa species that contains additional actin binding domains. Other studies have shown that MYLK-210 displays enhanced interaction with the actin cytoskeleton compared to the 130 kDa isoform [28,29]. These results are consistent with the enhanced migration of ONH astrocytes mediated in part by increased expression of MYLK-210.

MYLK variants have been found to confer risk of lung injury [30], asthma or sepsis [31], particularly in African Americans [32]. Some of the common polymorphisms in *MYLK *affect its expression [31]. Therefore, in some populations, it is possible that the effects of increased expression of *MYLK *may be further modified by genetic polymorphisms.

#### Actin-dependent astrocyte migration

From the microarray and qRT-PCR data the following genes were differentially expressed in AAG: *TGFBR2*, *TGFBR1*, *SMAD3*, *NCK1*, *PTPN11*, *ARHGEF7*, *PDLIM1*, *LM04*, and *PLEC1*. Figure 2a shows several signal transduction networks that participate in the regulation of actin. Remodeling or redistribution of actin at cellular edges is an essential part of establishing cell polarity [33] and the formation of processes in astrocytes [34]. Actomyosin interactions and actin polymerization are regulated by intracellular proteins such as α-actinin (ACTN4) and the ARP protein complex (ACTR2, WASP: Figure 2a). These networks involve the Rho GTPase signaling pathway. Therefore, we used a pull-down Rho activation assay to measure activated Rho in cell lysates. ONH astrocytes from CAG and AAG donors exhibited significantly higher Rho activity compared to those from normal AA and CA donors (Figure 2b, c), consistent with the differential expression of Rho regulatory components. Rho activity was also increased in astrocytes exposed to elevated hydrostatic pressure [35]. Thus, increased Rho activity is another contributor towards increased migration of AAG astrocytes. We suspect that Rho activity may be altered by changes in the signaling proteins included in these networks. For example, RAC2 and ARGEF7 are upregulated in AAG. The Rho-family GTPase, RAC2, is downstream of TGFβ signaling [36] and ARHGEF7 stimulates guanine nucleotide exchange on Rho family GTP-binding proteins. We further elaborated changes in TGFβ signaling as a driver to changes in Rho activity.

#### TGFβ signaling in AAG astrocytes

**Figure 5:**
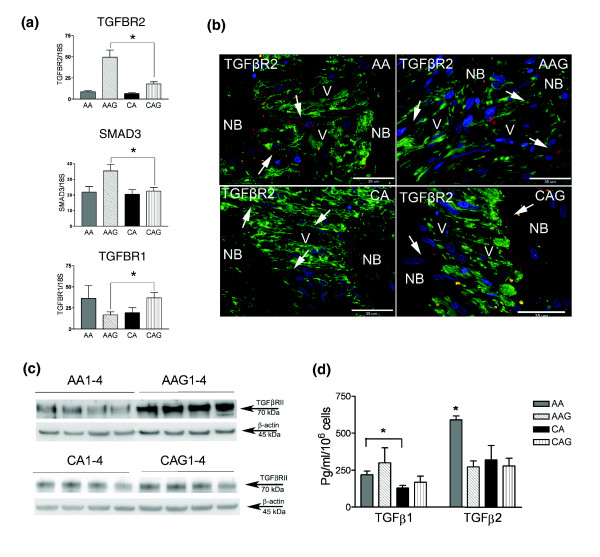
TGFβ and its receptors in ONH astrocytes. **(a) **Confirmation of three differentially expressed genes from the TGFβ-actin network (Figure 3a) by qRT-PCR in human ONH astrocytes: *TGFBR2*, *SMAD3 *and *TGFBR1*. Genes were normalized to 18S. Graphical representation of the relative mRNA levels in normal and glaucomatous AA and normal and glaucomatous CA astrocytes (n = 6, two-tailed *t*-test was used. Asterisk indicates *p *< 0.05). **(b) **Representative double immunofluorescent staining of TGFBR2 (red) and astrocyte marker GFAP (green) in sections of human ONH from an AA donor (51 year old male), AAG donor (70 year old male), CA donor (54 year old male) and CAG donor (76 year old male). Nuclei (blue) are stained with DAPI. Note granular staining of TGFBR2 in astrocytes (arrows) in the cribriform plates of the lamina cribrosa in AAG and CAG donors. Fewer astrocytes stain for TGFBR2 in the lamina cribrosa of CA donors. V, blood vessel; NB, nerve bundle. Scale bar 35 μm. **(c) **Representative western blots of astrocyte cell lysates with TGFBR2 antibody. β-Actin was used as a loading control. Note that AAG donors express more TGFBR2 than CAG donors. Normal AA and CA express lessTGFBR2 than glaucomatous donors. **(d) **Secreted TGFβ1 and TGFβ2 detected by ELISA. TGFβ2 is the primary form of TGFβ produced by ONH astrocytes. Secreted TGFβ1 is significantly higher in AA astrocytes compared to CA astrocytes (Asterisk indicates *p *< 0.05, two-tailed *t*-test); however, the increase in glaucomatous astrocytes compared to normal astrocytes is not significant. Secreted TGFβ2 levels are elevated significantly from normal AA astrocytes compared to all other donors (n = 24; asterisk indicates *p *< 0.05, two-tailed *t*-test).

TGFβ1 and TGFβ2 act via TGFBR1 and TGFBR2 receptors. Using qRT-PCR we confirmed that TGFBR2 and the downstream signaling protein SMAD3 are up-regulated in AAG astrocytes, suggesting increased responsiveness (Figure 5a). TGFBR1 is down-regulated in AAG compared to CAG (Figure 5a). SMAD proteins not only function as transcriptional regulators in ONH astrocytes [37] and other cells in the central nervous system [38], but also participate in the regulation of cell polarity. SMAD3 was also upregulated in ONH astrocytes exposed to hydrostatic pressure *in vitro*, suggesting that pressure activates the TGFβ pathway [35]. In addition, LM04, a LIM domain protein that modulates SMAD3 transcriptional activity [39], is upregulated in glaucomatous astrocytes in both populations (Table 1). One path that limits SMAD3 signaling is ubiquitin-linked degradation by SMURF2. Although SMURF2 expression is not altered in glaucomatous astrocytes, SMURF2 is downregulated by an increase in hydrostatic pressure [35]. Thus, there may be additional potentiation of TGFβ signaling in AAG astrocytes with changes in intraocular pressure, which may be a susceptibility factor to glaucomatous changes in the AA population.

TGFβ regulates cellular motility through two components. One is through the expression of extracellular matrix (ECM) proteins, which will be discussed in detail below. Contractile forces are transmitted to the ECM through actin-based stress fibers via focal adhesions, which are assemblies of ECM proteins, transmembrane receptors, and cytoplasmic structural and signaling proteins, such as integrins. TGFβ modulates integrin-mediated cellular migration, where FYN is one of the primary signal transducing proteins. A second component of TGFβ signaling is the regulation of cell polarity. For example, PARD3 and PARD6 are part of a multi-component polarity complex that controls polarized cell migration [40]. These complexes involve the Rho, CDC42, and RAC signaling pathways, which provide the means to remodel actin during migration [33,41]

As shown in Figure 2a, NCK1 was upregulated in AAG (Table 3). The Nck1 SH2/SH3 adaptor couples phosphotyrosine signals to the actin cytoskeleton and receptor signaling to the regulatory machinery of the cytoskeleton [42]. The enigma family member PDLIM1 was upregulated in AAG astrocytes (Table 3) and functions by allowing interactions among cytoskeletal proteins through PDZ and amino LIM domains [43,44]. Downregulation of other actin binding proteins such as PLEC1 (Table 3) may alter actin dynamics with respect to cytoskeletal changes induced by Rho-GTPase, phospholipids, and tyrosine kinase (Src) mediated signaling [45].

#### TGFBR2 receptors in optic nerve head astrocytes

Figure 5b illustrates immunohistochemistry of the TGFBR2 on astrocytes in normal and glaucomatous ONH tissue. GFAP positive astrocytes in the lamina cribrosa of AAG exhibit higher expression of TGFBR2 compared with astrocytes in normal ONH tissue. Consistent with these findings, western blots of lysates of ONH astrocytes from AAG indicate higher levels of TGFBR2 protein compared to the normal tissue and CAG (Figure 5c).

To further investigate alterations in TGFβ signaling in ONH astrocytes, we examined the production of TGFβ1 and TGFβ2. As seen in Figure 5d, TGFβ2 is the primary form of TGFβ produced by ONH astrocytes [46]. There are significantly increased levels of secreted TGFβ1 in AA compared to CA astrocyte supernatants but the increases in AAG and CAG astrocytes were not significant compared to normal astrocytes. These data suggest that most of the changes in TGFβ signaling are due to alterations at the level of TGFβ receptors in astrocytes from AAG.

Mutations in TGFBR2 are associated with Marfan syndrome type 2 [47-49]. Ocular abnormalities, including glaucoma, are associated with Marfan syndrome type 1 in which there are mutations in the gene for fibrillin (*FBN1*) [50]. However, it has not been established that mutations of TGFBR2 are associated with ocular problems in Marfan syndrome type 2 [48,49].

### Intracellular trafficking and the endoplasmic reticulum/Golgi compartments

From the microarray and quantitative RT-PCR data the following genes were differentially expressed in AAG. Endosome group, *RAB4A*, *RAB5B*, *RAB9P40*, *RAB9A*; plasma membrane group, *PRSS3*, *APPB1*, *CTNND1*, *CTNNB1*, *CDH2*, *VCAN*, *HAPLN1*, *CCL5*, *COL4A4*, *TGM2*, *SLIT2*, *GPC1*; Golgi group, *GOLGA1*, *GOLGA3*, *GOLGA2*, *RAB1A*, *RABGGTB *(Figure 3a). Six Rab family signaling genes involved in intracellular transport of organelles were differentially regulated (Table 3). Three small GTPases, RAB4A, RAB5B, and RAB9A, were upregulated (Table 3, Figure 3b), suggesting increased endosomal transport and processing. RAB4A and RAB5B selectively regulate intracellular trafficking and signaling of G protein-coupled receptors, such as the angiotensin receptor and adrenergic receptors (β2-AR and α2B-AR) from the cell surface [51,52]. RAB9A participates in late endosomal events leading to fusion with the lysosomal compartment [52].

In AAG astrocytes there was a predominant increase in transcription of Golgi-resident protein transcripts (Additional data file 7). These include *RAB1A*, and three members of the golgin family, *GOLGA1*, *GOLGA2 *and *GOLGA3 *(Table 3), which may function in the stacking of Golgi cisternae and in vesicular transport [53]. GOLGA3 promotes cell surface expression of the beta adrenergic receptors [54]. Thus, the increased expression of Golgi proteins may further enhance adrenergic receptor signaling. Note that the RAB proteins upregulated in the endosomal pathway (above) also affect trafficking of these receptors.

Included in the protein trafficking network are plasma membrane associated proteins involved in cell-cell communication from the junctional matrix (Figure 3a). Catenins (CTNNB1, CTNND1) form membrane trafficking complexes that integrate other cadherins (CDH2), and members of the amyloid precursor protein complex (presenilin, APPBP1, PRSS3). In particular, CTNND1 functions to regulate membrane trafficking either through blocking cadherin interactions, or through Rho-GTPases such as Rho A, Rac and CDC42 [55]. As with the myosin and actin motility networks, the change in expression of GTPase regulatory proteins will likely impact plasma membrane trafficking. The upregulation of chondroitin sulfate proteoglycan 2 (versican; *VCAN*), transglutaminase 2 (*TGM2*), and hyaluronan and proteoglycan link protein 1 (*HAPLN1*) are significant modifiers of the ECM [56]. Both *HAPLN1 *and *VCAN *mRNA levels were upregulated in AAG compared to CAG astrocytes by qRT-PCR (Figure 3b). VCAN immunoreactivity was observed in the ECM of the cribriform plates, the perivascular matrix and a few astrocytes in the lamina cribrosa of normal AA and CA donors (Figure 3c). In glaucomatous tissues there was a marked increase in VCAN staining in astrocytes in the cribriform plates and hypertrophied reactive astrocytes in the nerve bundles in both populations (Figure 3c). TGFβ2 signaling upregulates VCAN [57] in astrocyte cell types and expression of collagen type 4 and transglutaminase 2 in ONH astrocytes [37]. Our data on changes in TGFβ receptor expression and ECM proteins are similar to those found in microarray profiling of ONH tissue from a rat model of glaucoma [58]. Expression of ECM proteins is also modulated by TGFβ in GFAP-negative lamina cribrosa cells in culture [59].

There is substantial evidence that ONH astrocytes are responsible for the normal maintenance of the ECM in normal tissue and that reactive astrocytes remodel the ECM in response to elevated IOP in human and experimental glaucoma [10,60,61]. Reactive astrocytes in the ONH express abnormal ECM in glaucoma, leading to loss of resiliency and deformability in response to elevated IOP. Alterations in TGFβ2 levels and TGFβ receptors and abnormal synthesis of ECM in AAG may convey connective tissue components of susceptibility to elevated IOP to this population.

### cAMP signaling in glaucomatous ONH astrocytes

**Figure 6:**
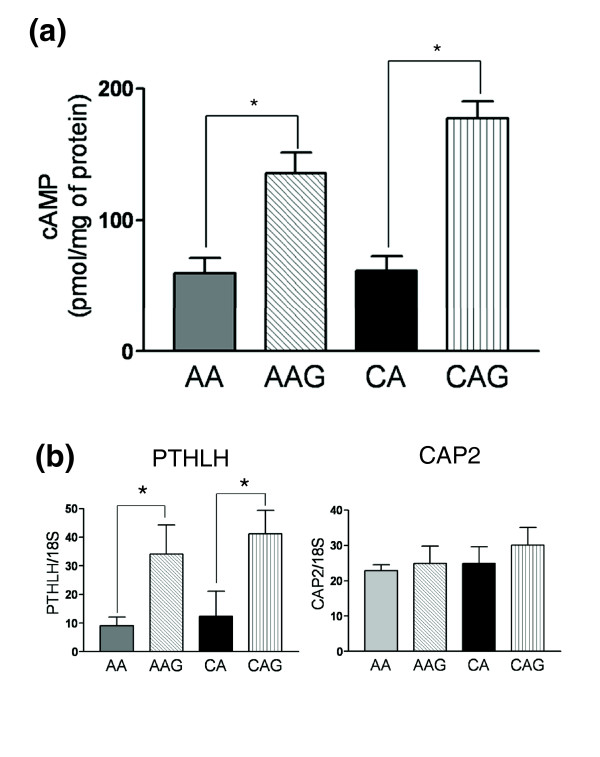
cAMP signaling in glaucomatous astrocytes. **(a) **cAMP levels in unstimulated ONH astrocytes were determined as described in the Materials and methods. The basal cAMP level was significantly higher in glaucomatous astrocytes compared to their normal counterparts. Values are the mean ± standard deviation of cAMP expressed in pmol/mg of protein. Eight AA, four AAG, nine CA and four CAG individual samples were used in this study. **(b) **Confirmation of *PTHLH *and *CAP2 *expression by qRT-PCR in human ONH astrocytes. Genes were normalized to 18S. Graphical representation of the relative mRNA levels in normal and glaucomatous AA and normal and glaucomatous CA astrocytes (n = 6, two-tailed *t*-test). Asterisk indicates *p *< 0.05).

Earlier work in our laboratory indicated upregulation of two adenylyl cyclases (ADYC3 and ADYC9) in normal AA compared to CA astrocytes, suggesting changes in cyclic AMP (cAMP) levels in this population (L Chen, MR Hernandez, ARVO (Association for Research in Vision and Ophthamology) 2007 abstract 3265). To test whether glaucomatous ONH astrocytes exhibit differential basal levels in cAMP, we conducted a standard cAMP assay in normal AA and CA astrocytes and in AAG and CAG astrocytes. Under unstimulated conditions, normal AA and CA astrocytes exhibit no difference in basal levels of cAMP, whereas AAG and CAG astrocytes have significantly higher basal levels of cAMP compared with the normal counterparts (Figure 6a). Cyclic AMP is a key intracellular second messenger in astrocytes. The cAMP signaling cascade opposes pro-inflammatory cytokines such as IL1β and TNFα and maintains astrocytes in a quiescent (non-activated) state [62]. Thus, the higher basal levels of cAMP in astrocytes from glaucomatous donors may be a response to pro-inflammatory cytokines such as TNFα in the glaucomatous ONH [19].

We searched the expression data for differentially expressed genes that might explain the difference in basal cAMP levels between glaucomatous and normal astrocytes. One potential candidate for increasing basal cAMP is PTHLH, a parathyroid hormone-like protein that is upregulated in glaucomatous astrocytes (Figure 6b). This protein binds to ubiquitous PTH receptors that are coupled to stimulation of adenylate cyclase and elevated cyclic AMP [63]. Thus, upregulation of PTHLH provides an autocrine pathway leading to increased basal cyclic AMP levels in glaucomatous astrocytes. Another gene that might also contribute to the activity of adenylate cyclases is CAP2 [64]. However, we found that CAP2 was not differentially expressed in glaucomatous ONH astrocytes by qRT-PCR (Figure 6b).

### Other disease-associated genes differentially regulated in glaucomatous OHN astrocytes

#### Cell-cell communication

**Figure 7:**
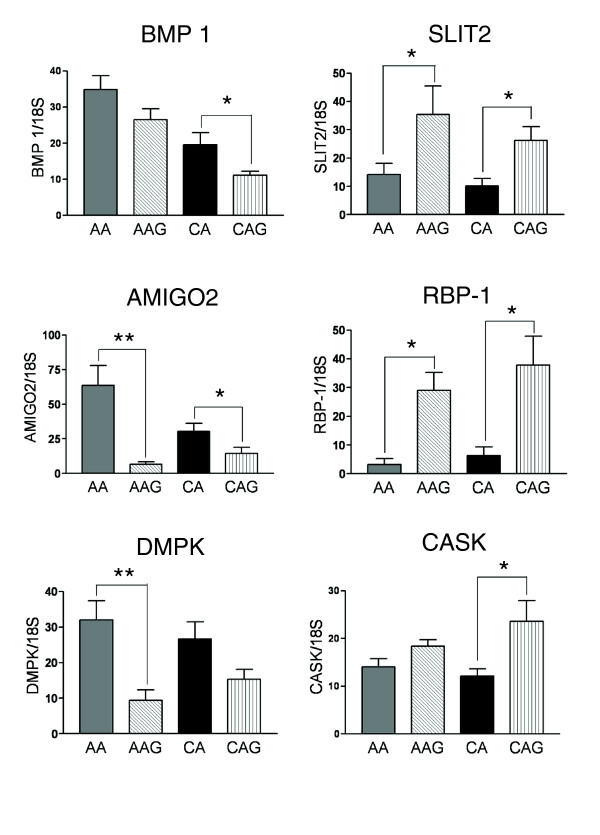
Glaucoma disease-associated genes differentially regulated in glaucomatous OHN astrocytes. Differential expression of six glaucoma disease associated genes (*BMP1*, *AMIGO2*, *DMPK*, *SLIT2*, *RBP-1 *and *CASK*) was validated by qRT-PCR in human ONH astrocytes. Genes were normalized to 18S. Graphical representation of the relative mRNA levels in normal and glaucomatous AA and normal and glaucomatous CA astrocytes (n = 6, two-tailed *t*-test). Asterisk indicates *p *< 0.05).

The secondary and tertiary comparisons identified genes that were differentially expressed in AAG compared to AA and in CAG compared to CA, including *BMP1*, *LTBP1*, *AMIGO2*, *SLIT2*, *GPC1*, and *OLR1 *(Tables 1 and 2). Selected genes were confirmed by qRT-PCR (Figure 7).

In this list we found that specific cell-surface-associated proteins are downregulated in glaucoma. These include BMP1, which activates cleavage of LTBP1 proteins that release nascent TGFβ1 [65], and AMIGO2, a type I transmembrane protein that regulates axon extension [66]. Down-regulation of BMP1 may reduce the levels of free TGFβ1 and thus unbalance signaling between TGFβ isoforms. A decrease in AMIGO2 might negatively impact axon survival.

Two differentially expressed genes that are involved in reactive astrocyte responses to neuronal injury are *SLIT2 *and *GPC1 *(glypican-1). SLIT2 serves as a chemorepellant for multiple types of axons [67], while GPC1 is a proteoglycan that binds SLIT2 [67]. Upregulation of expression of SLIT2 and a reduction of GPC1 by glaucomatous astrocytes suggest an inhibitory microenvironment for RGC axons in the ONH. These data are consistent with the idea that the enhanced migratory properties of glaucomatous astrocytes coupled with the release of factors that negatively impact upon axon survival are part of the pathophysiology of the disease.

Finally, lectin-like oxidized-LDL receptor (OLR1; also known as LOX-1) is highly upregulated (Additional data files 5 and 11) in AAG astrocytes. OLR1 expression is induced by TGFβ1 signaling and is known to be a component of the fluid shear stress response of endothelial cells in early atherosclerotic lesions [68]. These data are further confirmation of enhanced TGFβ signaling in AAG astrocytes as suggested by the differential receptor expression described earlier.

#### Intracellular calcium signaling/transport systems in ONH astrocytes

Two genes directly involved in Ca^2+ ^homeostasis are differentially regulated in ONH astrocytes of AAG (Additional data files 5 and 7). *CACNB4 *encodes a beta subunit of the voltage-dependent calcium channel complex. CACNB4 plays an important role in calcium channel function by modulating G protein inhibition, increasing peak calcium current, controlling the alpha-1 subunit targeting to the membrane and shifting the voltage dependence of activation and inactivation. The second gene, *ATP2C1 *(Additional data file 7), encodes a protein that belongs to the family of P-type primary ion transport ATPases, which pump Ca^2+ ^into the endoplasmic reticulum.

Transcripts encoding the calcium/calmodulin-related signaling proteins calmodulin 1 (CALM1) and Ca^2+^/calmodulin-dependent membrane-associated kinase (CASK1) are differentially expressed in one or more glaucoma groups. CALM1 was increased in AAG compared to AA donors (Additional data file 5), while CASK1 was increased in glaucomatous astrocytes from both AA and CA donors (Table 1, Figure 7). Calmodulin is the Ca^2+ ^sensor of key signaling molecules, such as adenylyl cyclase, CAMKII, CAMKIV, and MYLK discussed above. CASK1 is a member of the membrane-associated guanylate kinase proteins (MAGUKs), a prominent family of scaffolding molecules associated with intercellular junctions. CASK1 targets Ca^2+ ^and K^+ ^channels [69] and/or the Ca^2+ ^pump 4b/CI [70] to the plasma membrane, interacts with liprins [71] and regulates transcription by interacting with transcription factors in the nucleus [72]. Interestingly, *CASK *is a candidate gene for X-linked optic atrophy [73]. The differential expression of genes in Ca^2+ ^signaling pathways could be a common theme in glaucomatous astrocytes that may have a higher impact in optic nerves from AA donors due to increased sensitivity to elevated IOP in these donors.

## Conclusion

Glaucomatous ONH astrocytes share many characteristics of reactive astrocytes in the central nervous system; however, certain properties may be specific to the pathophysiology of glaucoma. The current work and previous studies demonstrate that cultured glaucomatous ONH astrocytes exhibit differential expression of genes that promote cell motility and migration, downregulate cell adhesion, are associated with structural tissue changes, and contribute to neural degeneration. Our data further strengthen the idea that reprogramming of transcription in glaucomatous astrocytes shifts signaling towards TGFβ, Rho GTPase and Ca^2+ ^systems, which impact the multiple networks described earlier.

Our demonstration of this wide variety of genes that remain differentially expressed after weeks in culture suggests that glaucomatous ONH astrocytes have an altered phenotype. In the current study, using microarray analysis, we identified a number of genes (for example, *MYLK*, *TGFBR2*, *VCAN*, and *RAC2*) whose expression may underlie higher susceptibility of astrocytes of AA individuals to elevated IOP and that may be relevant to reactive astrocyte responses in glaucoma. Some limitations of our approach should be noted. First, ONH astrocyte derived from human glaucomatous eyes during the disease process does not allow assessment of changes or the identification of early mechanisms of disease that might be available from animal models. In addition, the difficulty to obtain and include more AA glaucomatous eyes limited our ability to identify differentially expressed genes in this group. However, stringent filters allowed the selection of a group of genes with functional significance. For each comparison, selected genes were validated by qRT-PCR and relevant gene products were confirmed by western blots in the four groups.

We propose that part of the increased susceptibility to elevated IOP in AAG relates directly to astrocyte functions in the ONH. Astrocytes in AAG, which are reactive astrocytes, may have increased responsiveness to TGFβ signaling and enhanced migratory abilities, which may impact the remodeling of the ECM, inhibit axon survival, and alter vascular permeability in the glaucomatous ONH. Any one of these changes may represent a susceptibility risk factor in the AA population to withstand abnormally elevated IOP.

This study provides an initial survey of the molecular differences of ONH astrocytes from AA and CA donors with glaucoma. Genes encoding many potential therapeutic targets, such as motility genes, ion channels, adhesion molecules, and signaling pathways, are selectively expressed in glaucomatous astrocytes, making them interesting as potential targets for astrocyte-specific therapeutics. Additional applications of these data include identification and characterization of signaling pathways involved in astrocyte function and further exploration of the role of selected identified genes in experimental animal and in *vitro *models of glaucoma.

## Materials and methods

### Human eyes

Thirteen eyes from eleven CA donors (age 73 ± 9 years) with POAG (referred to as CAG) and six eyes from three AA donors (age 62 ± 13 years) with POAG (referred to as AAG) were used to generate ONH astrocyte cultures as described (Additional data file 1). Myelinated optic nerves were fixed in 4% paraformaldehyde, post-fixed in osmium, embedded in epoxy resin and stained with paraphenylendiamine to detect axon degeneration as described earlier to confirm glaucoma and to assess optic nerve damage (Additional data file 1). Normal eyes were from 12 CA donors (age 60 + 11 years) and 12 AA donors (age 58 + 12 years) with no history of eye disease, diabetes, or chronic central nervous system disease (Additional data file 2).

### Astrocyte cultures

Primary cultures of human ONH astrocytes were generated as described previously [11]. Briefly, four explants from each lamina cribrosa were dissected and placed into 25 cm^2 ^Primaria tissue culture flasks (Falcon, Lincoln Park, NJ, USA). Explants were maintained in DMEM-F12 supplemented with 10% fetal bovine serum (Biowhittaker, Walkerswille, MD, USA) and 10 μl/ml of PSFM (10,000 U/ml penicillin, 10,000 μg/ml streptomycin and 25 μg/ml amphotericin B; Gibco/BRL, Gaithersburg, MD, USA). Cells were kept in a 37°C, 5% CO_2 _incubator. Primary cultures were purified by using an immunopanning procedure [11]. Purified cells were expanded after characterization by immunostaining for astrocyte markers GFAP and NCAM (Neural cell adhesion molecule) as described [11]. Second passage cell cultures were stored in RPMI 1640 with 10% DMSO in liquid nitrogen until use. For each set of experiments, cells were thawed and cultured so that sufficient cells from the same batch were available for multiple experiments.

### Antibodies

An affinity purified rabbit polyclonal antibody to MYLK was a gift from Dr Linda van Eldik (Northwestern University). It was used in western blotting (1:10,000) and immunohistochemistry (1:50). Another MYLK antibody (M7905) is a mouse monoclonal antibody (Sigma-Aldrich, St Louis, MO, USA) and it was used in western blotting (1:10,000) and immunohistochemistry(1:50). TGFBRII (L-21) is a rabbit polyclonal antibody (Santa Cruz Biotechnology Inc., Santa Cruz, CA, USA). It was used in western blotting (1: 200) and immunohistochemistry (1:50). VCAN is a goat polyclonal antibody (R&D Systems, Minneapolis, MN, USA). It was used in immunohistochemistry (1:20).

### Oligonucleotide microarray analysis

Total RNA was extracted using Qiagen RNeasy mini kits (Qiagen, Valencia, CA, USA). RNA was then purified and quantified by measuring absorbance at 260 nm. Quality and intactness of the RNA was assessed by capillary electrophoresis analysis using an Agilent 2100 Bioanalyzer (Agilent, Palo Alto, CA, USA). cDNA was synthesized from 2-5 μg purified RNA by using Superscript Choice system (Gibco BRL Life Technologies, Gaithersburg, MD, USA) and T7-(dT)24 primer (GENSET, La Jolla, CA, USA). Using Bioassay High Yield RNA Transcript Labeling Kit (Enzo Diagnostics, Farmingdale, NY, USA), *in vitro *transcription was carried out with the cleaned double-stranded cDNA as a template in the presence of biotinylated UTP and CTP. Purified biotin-labeled cRNA was fragmented before the hybridization. Hybridization of the labeled cRNA to Human Genome U95Av2, U133A, U133A 2.0 chips (Affymetrix, Santa Clara, CA, USA) was carried out by using Genechip Instrument System (Affymetrix) at the Genechip Core Facility of Washington University School of Medicine. The arrays were washed and stained with streptavidin-phycoerythrin (Molecular Probes, Eugene, OR, USA) followed by scanning on an Agilent GeneArray Scanner G2500A (Agilent Technologies, Palo Alto, CA, USA).

### Data analysis

#### Pretreatment of data

The first step in the analysis of the microarray data was to determine which genes to consider 'present' or 'absent'. We estimated the probe-set present/absent calls by using the Wilcoxon signed rank-based algorithm. In order to reduce false positives, we removed the probe-sets with all samples as 'absent' (Additional data file 3).

#### Comparison between glaucomatous ONH astrocytes from AA and CA normal donors

As the experiments were done at different times, two types of Affymetrix microarrays (Human Genome U95Av2 array and Human Genome U133A 2.0 array) were used. Samples from eight CAG donors, seven CA normal donors and six AAG samples were measured using a Human Genome U95Av2 array. Eighteen CA samples, eighteen AA samples and six AAG samples were measured using a Human Genome U133A 2.0 array. The data measured by two types of arrays were normalized separately by RMA normalization [74,75]. We defined common glaucoma-related genes as genes differentially expressed in both CAG versus CA and AAG versus AA, and did comparisons of CAG versus CA and AAG versus AA separately. The differentially expressed genes were identified by the empirical Bayesian shrinkage moderated t-statistics in the *limma *Bioconductor package [76]. A mixed effects model was used to account for the effect of technical replicates. Genes exhibiting a fold-change >1.5 and *p*-value < 0.01 were considered significant. To reduce false positives because the AAG has only three biological replicates, we applied the Benjamini and Hochberg false discovery rate multiple testing correction with a false discovery rate of 0.05 (AAG versus CAG and AAG versus AA).

To compare the significant gene list based on two types of microarray platforms, the Affymetrix probeset IDs were transferred as Entrez IDs based on the Bioconductor library. Genes whose Entrez IDs appear in both the differentially expressed gene list from CAG versus CA (using the Human Genome U95Av2 array) and AAG versus AA comparisons (using the Human Genome U133A 2.0 array) and change in the same direction were considered as common glaucoma-related genes. Genes that are differentially expressed for AAG versus CAG (using the Human Genome U95Av2 array), but without significant changes for AA versus CA (using Human Genome U133A 2.0 array), were considered as the glaucoma race-related genes. Here we considered a *p*-value > 0.05 as indicative of changes that were not significant.

GO analysis of differential expression in glaucomatous astrocytes was done with GoMiner [14]. Briefly, gene lists of up- and downregulated genes (*p *< 0.01 as described above) were normalized to 1 and -1, respectively, for genes that exhibited at least a 1.5-fold change in either direction. These lists were then loaded into GoMiner using local GO databases accessed using the 'Derby' module. GoMiner output was analyzed with a significance cutoff of *p *< 0.01 and at least four genes per category.

### Network construction

Initially, we scanned the differentially expressed gene lists for AAG-CAG, AAG-AA, and CAG-CA comparisons for groups of genes that were either in common GO categories, or were highly over- or underexpressed (>1.5-fold, *p *< 0.01). These short lists were then used as a source of nodes for each network group. Networks of interacting proteins were constructed using the BioGRID database [77]. BioGRID is a freely accessible database of physical and genetic interactions. BioGRID release version 2.0 includes more than 116,000 interactions from *Saccharomyces cerevisiae*, *Caenorhabditis elegans*, *Drosophila melanogaster *and *Homo sapiens*. Graphs with embedded protein, gene and interaction attributes were constructed with a visualization program, Osprey [78], that is dynamically linked to the BioGRID. Each network was begun using a single gene or node. Then more interactions were added using The BioGrid Database lookup function. These were curated to simplify the graphs, and non-expanded nodes were minimized. In general, nodes that were not differentially expressed required at least two connections or edges to remain in the network. Expression of genes depicted in the networks were checked for a 'present call' in the microarray data or otherwise validated by quantitative real time RT-PCR.

### Real-time qRT-PCR

Real time qRT-PCR was done as previously described [60]. To compare expression of specific genes amongst the four groups included in this study (AA, CA, AAG and CAG), we used 12 ONH astrocyte cultures from normal CA and 12 cultures from normal AA donors. cDNA of eight eyes from eight CAG donors and of six eyes from three AAG donors were used. Individual samples were processed simultaneously under the same conditions and the data were analyzed for significance using a two-tailed *t*-test on sample pairs (Prism 3.0 GraphPad software, San Diego, CA, USA). Primers used in this study are listed in Additional data file 4.

### Western blotting

Protein lysates from four samples of each group of ONH astrocytes were processed together in the appropriate combinations: four AAG and four AA; four CA and four CAG. Western blots were run in triplicate to accommodate all available samples. Protein lysates containing 3-10 μg were used depending on the specific antibody. β-Actin was used as a loading control. Films of blots were scanned using a flatbed scanner in 8-bit gray scale mode. ImageJ (National Institutes of Health) was used to quantify band intensities on the blots.

### Detection of TGFβ1 and TGFβ2 by ELISA

TGFβ1 and TGFβ2 were measured in cell culture supernatants using ELISA kits (R & D Systems) specific for each protein. Briefly, astrocytes (70-80% confluent) were incubated for 24 h in 6 ml of cell culture medium without serum. Media was harvested and divided into 1 ml aliquots and frozen at -80°C until analysis. For each sample, cell counts were made and recorded. Media samples were thawed on ice and 200 μl aliquots activated by incubating with 40 μl of 0.1 N HCl at room temperature for 40 minutes. The reactions were quenched by adding 40 μl of 0.1 N NaOH in 0.5 M HEPES and mixed. Samples were diluted with the appropriate ELISA assay buffer to 400 μl. Aliquots of these solutions (50 μl TGFβ1:100 μl TGFβ2) were then assayed according to the manufacturers' protocol. Experiments were performed in duplicate and each astrocyte cell culture (n = 5-7 samples per each group) was assayed at least twice. Expressed protein values in picograms of TGFβ1/2 per ml were normalized to 10^6 ^cells using the cell counts obtained at harvest. The means of the content were considered significantly different if *p *< 0.05 (two-tailed *t*-test; Prism 3.0 GraphPad software.).

### Cyclic AMP assay

Primary ONH astrocyte cultures obtained from six normal AA, six normal CA, eight CAG and three AAG were grown in 60 mm dishes until 80% confluence. Growth media was replaced with serum free media and the cells incubated for an additional 24 h. After washing with ice-cold phosphate-buffered saline (PBS), cells were lysed in 95% chilled ethanol for 1 h and then centrifuged at 2000 × g for 15 minutes at 4°C. The supernatant was evaporated using a Speed Vac concentrator and resuspended in 100 μl of the assay buffer and analyzed as described in the cAMP Biotrak Enzyme Immunoassay Kit (Amersham Bioscience RPN225, Piscataway, NJ, USA). cAMP concentration per well was expressed as pmol/mg of protein. Each value represents the mean cAMP level (± standard deviation) of independent experiments using primary astrocyte cultures from each donor and performed in triplicate. Sample pairs were analyzed by two-tailed *t*-test (Prism 3.0 GraphPad software) for significance (*p *< 0.05).

### Migration assay

CytoSelect™ 24-well cell migration assay (Cell BioLabs, San Diego, CA USA) was used to measure the migratory properties of cells. The assay was performed according to the manufacture's protocol. Briefly, media with 10% fetal bovine serum was placed in the lower wells followed by 50,000 cells in 300 μl of serum free media in each of the well inserts. After incubation at 37°C in a 5% CO_2 _atmosphere for 24 h, the media was removed from the inserts. Cells that did not migrate were removed from the inserts using a cotton swab. The inserts were stained with 400 μl of cell staining solution and washed three times with water. Cells were treated with 200 μl of extraction solution and the solution transferred to individual wells of a new plate. The absorbance of the extracted samples was measured at 560 nm by a Thermo Multiskan Spectrum plate reader. Six astrocyte cultures from each group (AA, CA, AAG and CAG) were used in the assay and data were analyzed for significance with ANOVA (Prism 3.0 GraphPad software).

### Rho activation assay

Rho activation assay kit (Upstate Biotechnology Billerica, MA, USA) was used to detect activated Rho in cell lysates. Unstimulated cells were cultured in 60 mm dishes until 85-90% confluence and then harvested in ice cold 1 × Mg^2+ ^Lysis/Wash (MLB) buffer (according to the manufacturer's protocol). Protein concentration was determined by the Bradford method. Protein lysate (200 μg) were mixed with 32 μl of Rho assay reagent slurry containing GST-Rhotekin-RBD fusion protein, and incubated for 45 minutes at 4°C with gentle agitation. After pelleting and washing three times with 1 × MLB, the beads were resuspended in 2 × NuPage LDS sample buffer (Invitrogen Carlsbad, CA, USA) supplemented with 0.075 M DTT and boiled at 95°C for 5 minutes. Samples were subjected to western blot analysis. An anti-Rho antibody that recognizes Rho-A, Rho-B and Rho-C was used for detection. Four cultures from each group (AA, CA, AAG and CAG) were used in the assay. Western blots were performed in duplicate. Representative blots are shown in the results and the mean optical density was used in density analysis. Statistical significance was based upon two-tailed *t*-test (Prism 3.0 GraphPad software) and *p*-value < 0.05

### Immunohistochemistry

Six eyes from normal CA donors, six eyes from normal AA donors, six eyes from CAG donors and four eyes from AAG donors were used. All donors were age matched. Tissues were fixed with 4% paraformaldehyde in 0.1 M phosphate-buffered saline pH 7.4 and processed for paraffin embedding. Two slides were stained per donor containing at least two 6 μm optic nerve sections each. In double labeling experiments we used monoclonal or polyclonal antibodies against human glial acidic fibrillar protein (GFAP) as an astrocyte marker. Secondary antibodies labeled with Alexa 488 and Alexa 568 (1:800) were from Molecular Probes. For negative controls, the primary antibody was replaced with non-immune serum. Serial sections used in comparisons (AAG versus CAG) were stained simultaneously to control for variations in immunostaining. Slides were examined in a Nikon Eclipse 80 *i *microscope (Tokyo, Japan) equipped with epifluorescent illumination and digital cameras (CoolSnap ES and CF, Photometrics). The images were processed using MetaMorph software (Molecular Devices Sunnyvale, CA, USA).

## Abbreviations

AA, African American; AAG, AA donor with glaucoma; CA, Caucasian American; CAG, CA donor with glaucoma; cAMP, cyclic AMP; ECM, extracellular matrix; ELISA, enzyme-linked immunosorbent assay; GFAP, glial acidic fibrillar protein; GO, Gene Ontology; IOP, intraocular pressure; MYLK, myosin light chain kinase; ONH, optic nerve head; POAG, primary open angle glaucoma; qRT-PCR, quantitative RT-PCR; TGF, transforming growth factor; TNF, tumor necrosis factor.

## Authors' contributions

MRH conceived the study, directed individual efforts, and wrote drafts of the manuscript. TJL performed network analysis, data mining, and wrote drafts of the manuscript. HM coordinated molecular biology studies, cultured cell preparations, and contributed sections of the manuscript. LC and WL performed molecular biology and biochemical analyses. SMR, AMC, and AW performed migration assays and cell/tissue immunohistochemistry experiments. SNL and PD performed statistical analysis and bioinformatics on the microarray data. All authors viewed and approved the manuscript.

## Additional data files

The following additional data files are available in the online version of the paper. Additional data file 1 is a table listing clinical information about CAG and AAG eyes used to generate primary cultures of ONH astrocytes. Additional data file 2 is a table listing demographic information of CA and AA normal donor eyes used to generate primary cultures of ONH astrocytes. Additional data file 3 is a table that summarizes the number of probe-sets on the chip and used in analysis. Additional data file 4 is spreadsheet listing the primers used for qRT-PCR. Additional data file 5 is a spreadsheet listing genes differentially expressed in glaucomatous ONH astrocytes and including the comparison between AAG versus normal AA. Additional data file 6 is a spreadsheet listing differentially expressed genes between CAG and normal CA. Additional data file 7 is a spreadsheet listing differentially expressed genes between AAG and CAG. Additional data file 8 is a spreadsheet listing genes differentially expressed in ONH astrocytes from AAG compared to both normal AA and CAG. Additional data file 7 is a spreadsheet summarizing Gene Ontology for the comparisons between AAG and AA data. Additional data file 8 is a spreadsheet with Gene ontology comparisons for CAG and CA. Additional data file 9 is a spreadsheet with GO comparisons for AAG versus CAG expression sets. Additional data file 10 is a figure showing the distribution of genes in two GO categories. Additional data file 11 is a figure showing qRT-PCR data that confirm additional differentially expressed genes from the CAG-CA comparison.

## Supplementary Material

Additional data file 1 Clinical information about CAG and AAG eyes used to generate primary cultures of ONH astrocytes. Click here for file

Additional data file 2 Demographic information of CA and AA normal donor eyes used to generate primary cultures of ONH astrocytes. Click here for file

Additional data file 3 Probe-sets on the chip and used in analysis. Click here for file

Additional data file 4 Primers used for qRT-PCR. Click here for file

Additional data file 5 Includes the comparison between AAG versus normal AA. Click here for file

Additional data file 6 Differentially expressed genes between CAG and normal CA. Click here for file

Additional data file 7 Differentially expressed genes between AAG and CAG. Click here for file

Additional data file 8 Differentially expressed genes in ONH astrocytes from AAG compared to both normal AA and CAG. Click here for file

Additional data file 9 GO comparisons for AAG versus CAG expression sets. Click here for file

Additional data file 10 Common genes were selected from the GO lists (Additional data files 7-9) for each dataset (AAG-CAG, AAG-AA, and CAG-CA comparisons). The fraction of common genes (y-axis) for the GO terms 'phosphorylation' (grey bar) and 'cell-cell signaling' categories (black bar) are shown. Click here for file

Additional data file 11 **(a-e) **CAG-CA and comparison: CALM (a), CAPG (b), GJA1 (c), GPNMB (d) and SOD2 (e). **(f-j) **AAG-AA comparison: GSTA4 (f), LOXL2 (g), MYH10 (h), PDLIM7 (i) and OLR1 (j). Genes were normalized to 18S. Graphical representation of the relative mRNA levels in normal and glaucomatous AA and normal and glaucomatous CA astrocytes (n = 6, two-tailed *t*-test was used. Asterisk indicates *p *< 0.05). Click here for file
