# Supplementary material for: Susceptibility to glaucoma: differential comparison of the astrocyte transcriptome from glaucomatous African American and Caucasian American donors
Source: Genome Biol. 2008 Jul 9;9(7):R111. doi: 10.1186/gb-2008-9-7-r111 (PMC2530868; doi:10.1186/gb-2008-9-7-r111)
Supplement: Additional data file 1 — Clinical information about CAG and AAG eyes used to generate primary cultures of ONH astrocytes. [file gb-2008-9-7-r111-S1.doc]

| ***Table S1A: Clinical information of Caucasian American donor eyes with POAG used to generate culture of ONH astrocytes*** | | | | | | | | | |
| --- | --- | --- | --- | --- | --- | --- | --- | --- | --- |
|  | | | | | | | | | |
| **Donor** | **Gender** | **Age** | **Medical history** | **COD** | **TOD** | **TOE** | **Severity of glaucoma** ^#^ | **Glaucoma treatment** | **Experiments** |
| **00-6L** | F | 56 | CVA, SAH | SAH | 5:21 | 13:45 | Mild | Latanoprost | MA, RT-PCR, WB |
| **00-6R** | F | 56 | CVA, SAH | SAH | 5:21 | 13:45 | Mild | Latanoprost | MA, RT-PCR, WB, ELISA, FA |
| **00-7L** | M | 83 | CAD, HTN | CHF | 6:00 | 12:00 | Moderate | Xalatan | MA, RT-PCR, FA |
| **00-7R** | M | 83 | CAD, HTN | CHF | 6:00 | 12:00 | Moderate | Xalatan | MA, RT-PCR, WB, ELISA, FA |
| **02-1R** | M | 69 | CHF | ARF | 6:59 | 11:00 | Moderate | None | MA, RT-PCR, WB |
| 02-7L | M | 71 | HTN | CVA | 23:50 | 3:55 | Moderate | None | WB, RT-PCR, ELISA, FA |
| **02-10R** | F | 80 | Pneumonia | ARF | 6:00 | 12:00 | Advanced | None | MA, RT-PCR, WB |
| **04-1L** | F | 79 | COPD, CHF | CHF | 21:15 | 7:35 | Mild | None | MA, RT-PCR, ELISA, FA |
| **04-5L** | F | 72 | CHF, MI | CHF, RF | 5:22 | 10:35 | Moderate | None | MA, RT-PCR, ELISA, FA |
| 04-16L | M | 80 | CHF, MI | CHF | 23:30 | 2:30 | Advanced | Alphagan, Cosopt | RT-PCR, ELISA, FA |
| 05-2R | M | 70 | Prostate cancer | Cardiac arrest | 1:18 | 4:15 | Mild | Xalatan | RT-PCR, WB |
| 05-12R | F | 77 | Pancreatic cancer | Pancreatic cancer | 21:22 | 23:55 | Mild | Xalatan, Alphagan, Timolol | RT-PCR, WB, ELISA |
| 05-15R | M | 61 | Colon cancer | Renal failure | 21:53 | 23:20 | Mild | Eye drops** | WB, RT-PCR |

| ***Table S1B:*** ***Clinical information of African American donor eyes with POAG used to generate culture of ONH astrocytes*** | | | | | | | | | |
| --- | --- | --- | --- | --- | --- | --- | --- | --- | --- |
|  | | | | | | | | | |
| **Donor** | **Gender** | **Age** | **Medical history** | **COD** | **TOD** | **TOE** | **Severity of glaucoma #** | **Glaucoma treatment** | **Experiments** |
| **04-20L** | M | 67 | Stroke, CHF, HTN, CRF | Cardiac arrest | 2:00 | 4:40 | NA | None* | MA, RT-PCR, WB, ELISA, FA |
| **04-20R** | M | 67 | Stroke, CHF, HTN, CRF | Cardiac arrest | 2:00 | 4:40 | NA | None | MA, RT-PCR, WB, ELISA, FA |
| **04-24L** | F | 53 | Sarcoidosis, HTN | Cardiac arrest | 18:30 | 0:25 | Advanced | None | MA, RT-PCR, WB, ELISA, FA |
| **04-24R** | F | 53 | Sarcoidosis, HTN | Cardiac arrest | 18:30 | 0:25 | Moderate | None | MA, RT-PCR, WB, ELISA, FA |
| **05-16L** | F | 56 | Gastric cancer | Gastric cancer | 15:34 | 20:50 | Moderate | None | MA, RT-PCR, WB, ELISA, FA |
| **05-16R** | F | 56 | Gastric cancer | Gastric cancer | 15:34 | 20:50 | Moderate | None | MA, RT-PCR, WB, ELISA, FA |

^#^Severity of glaucoma was based on evaluation of the myelinated optic nerve and clinical ophthalmic history. COD: cause of death; TOD: time of death; TOE: time of enucleation. ARF: acute respiratory failure; CAD: coronary artery disease; CHF: chronic heart failure; COPD: chronic obstructive pulmonary disease; CRF: chronic renal failure; CVA: cerebral-vascular accident; GC: gastric cancer; HTN: hypertension. MI: Myocardial infarction, RF: renal failure, SAH: spontaneous aneurysm hemorrhage. None*: No treatment indicated in the charts. Eye drops**: indicated in the chart. MA: microarray, WB: western blot, FA: functional assay including proliferation assay, cAMP assay, adhesion assay, migration assay and Rho activation assay. Donors in **bold** were used for microarray analysis.

**Evaluation of Nerve Damage**

To evaluate optic nerve damage in eyes with clinical diagnostic of glaucoma, cross sections were taken from the myelinated optic nerves and stained with *p*-phenylenediamine (Pena et al 1998; 2001). Digital images were taken with at 2X magnification, so that the entire circumference of the nerve was within the lens field. Images were imported into Optimas software, where the total circumference area as well as the areas with axonal degeneration was measured. Results were expressed as a ratio between area of axon degeneration and total area. “Mild” axon loss was defined as a loss of up to one third of myelinated axon area, “moderate” axon loss when there was loss between one and two thirds of myelinated axon area, and “marked” axon loss when the loss in axon area surpassed two thirds of the total myelinated area.
