# Supplementary material for: Susceptibility to glaucoma: differential comparison of the astrocyte transcriptome from glaucomatous African American and Caucasian American donors
Source: Genome Biol. 2008 Jul 9;9(7):R111. doi: 10.1186/gb-2008-9-7-r111 (PMC2530868; doi:10.1186/gb-2008-9-7-r111)
Supplement: Additional data file 2 — Demographic information of CA and AA normal donor eyes used to generate primary cultures of ONH astrocytes. [file gb-2008-9-7-r111-S2.doc]

***Table S2: Demographic information of normal donors***

| **Donor** | **Cell ID** | **Gender** | **Age** | **Medical history** | **COD** | **TOD** | **TOE** | **Experiments** |
| --- | --- | --- | --- | --- | --- | --- | --- | --- |
| CA1 | **LC76** | F | 76 | HTN | CVA | 4:00 | 10:35 | MA, RT-PCR |
| CA2 | **99-9R-L** | F | 46 | Heart disease | Myocardial infarction | 0:37 | 5:45 | MA, RT-PCR, WB, ELISA |
| CA3 | **99-6L** | M | 42 | None | Head trauma | 21:06 | 23:30 | MA, RT-PCR |
| CA4 | **01-1R** | M | 52 | Hypotension, syncope | CHF | 6:25 | 9:40 | MA, RT-PCR, FA |
| CA5 | **03-11R** | F | 68 | Heart disease, bronchiectasis | Sepsis | 16:20 | 20:30 | MA, RT-PCR |
| CA6 | **03-13L** | F | 72 | Heart disease, HTN | Myocardial infarction | 20:50 | 1:52 | MA, RT-PCR, WB, FA |
| CA7 | **03-1L** | M | 49 | Hypotension | Metastatic esophageal cancer | 4:25 | 8:35 | MA, RT-PCR, WB, ELISA, FA |
| CA8 | **04-19L** | M | 50 | Heart disease, HTN | Aspiration pneumonia | 13:56 | 18:00 | MA, RT-PCR, FA |
| CA9 | **04-2R-L** | M | 65 | Heart disease, HTN | Cardiac arrest | 1:01 | 5:15 | MA, RT-PCR, WB, ELISA, FA |
| CA10 | **04-2R** | M | 65 | Heart disease, HTN | Cardiac arrest | 1:01 | 5:15 | MA, RT-PCR, WB, ELISA, FA |
| CA11 | **04-4L** | M | 42 | Liver carcinoma | Hypotension, cancer | 6:21 | 9:30 | MA, RT-PCR, WB, ELISA, FA |
| CA12 | **04-6L** | M | 57 | HTN, cancer of mouth and throat | Cardiac arrest | 3:57 | 9:55 | MA, RT-PCR, WB, FA |
| CA13 | **05-11R** | M | 68 | Anemia, cirrhosis | Intracranial bleeding | 3:15 | 6:00 | MA, RT-PCR, ELISA, FA |
| CA14 | **05-5L** | M | 56 | COPD | Cardiac arrest | 22:50 | 2:25 | MA, RT-PCR, WB, ELISA, FA |
| CA15 | **LC46** | M | 46 | CVA | Cerebrovascular/stroke | 17:42 | 2:45 | MA, RT-PCR |
| CA16 | 02-8R | F | 73 | Heart disease, renal insufficiency | CHF | 0:40 | 9:25 | MA, RT-PCR |
| CA17 | 03-17L | M | 56 | Lung cancer | Cardiac arrest | 21:18 | 2:30 | RT-PCR, ELISA, FA |
| CA18 | 04-22R | F | 77 | Renal failure, heart disease | Renal disease | 19:30 | 22:03 | RT-PCR, FA |
| CA19 | 04-23R | F | 78 | Subarachnoid hemorrhage | CVA | 16:31 | 20:50 | RT-PCR, ELISA |
| CA20 | 04-3L | M | 64 | Colon cancer, HTN | Myocardial infarction | 21:51 | 3:45 | FA |
| CA21 | 04-7R | M | 58 | HTN | Cardiopulmonary arrest | 17:24 | 1:20 | ELISA, FA |
| CA22 | 07-1L | M | 83 | Heart disease, HTN | COPD | 8:50 | 14:45 | WB, ELISA |
| CA23 | 07-10L | M | 72 | Skin cancer, arthritis | Full arrest | 17:46 | 1:11 | ELISA |
| AA1 | **02-3L** | F | 70 | HTN, CHF | Multi system organ failure | 5:46 | 13:34 | MA, RT-PCR, WB, ELISA, FA |
| AA2 | **02-5R** | M | 43 | HTN | Gun shot wound | 0:02 | 3:45 | MA, RT-PCR, WB, ELISA |
| AA3 | **02-6L** | M | 64 | Prostate cancer, heart disease | Myocardial infarction | 6:45 | 11:35 | MA, RT-PCR, FA |
| AA4 | **03-19L** | M | 56 | Sarcoidosis, HTN | Cardiopulmonary arrest | 17:26 | 23:40 | MA, RT-PCR, ELISA, FA |
| AA5 | **03-6R** | M | 45 | Heart disease | Cardiac arrest | 16:00 | 20:00 | MA, RT-PCR, WB, ELISA, FA |
| AA6 | **04-15L** | F | 70 | HTN, myocardial infarction | Cardiopulmonary arrest | 20:29 | 4:00 | MA, RT-PCR |
| AA7 | **04-21R** | M | 57 | HTN, lung cancer | Metastatic lung cancer | 22:50 | 4:00 | MA, RT-PCR, WB, ELISA, FA |
| AA8 | **05-13L** | M | 73 | COPD, lung cancer | Metastatic lung cancer | 23:00 | 5:00 | MA, RT-PCR, ELISA, FA |
| AA9 | **05-14L** | M | 38 | Bowel surgery due to stabbing | Blunt force trauma | 15:49 | 21:40 | MA, RT-PCR, FA |
| AA10 | **05-4L** | F | 47 | Abdominal surgery | Intracerebral hemorrhage | 4:42 | 9:10 | MA, RT-PCR, WB, ELISA, FA |
| AA11 | **05-7L** | M | 70 | Bladder cancer, metastatic lung cancer | Bladder cancer | 22:44 | 4:25 | MA, RT-PCR, ELISA, FA |
| AA12 | **05-8R** | M | 62 | COPD, HTN | Pulmonary embolism | 0:45 | 7:55 | MA, RT-PCR, ELISA, FA |
| AA13 | 03-12R | F | 73 | Heart disease | Myocardial infarction | 11:29 | 13:45 | FA |
| AA14 | 03-22R | F | 65 | Arthritis | Lung cancer | 23:00 | 3:35 | RT-PCR |
| AA15 | 03-9R | M | 68 | HTN | Cardiac arrest | 14:06 | 23:58 | FA |
| AA16 | 06-4L | M | 58 | Cardiomyopathy, CHF, HTN | Cardiac arrest | 19:55 | 2:00 | WB |

CA: Caucasian; AA: African American; COD: cause of death; TOD: time of death; TOE: time of enucleation. COPD: chronic obstructive pulmonary disease; CHF: congestive heart failure; CVA: cerebrovascular accident; HTN: hypertension; NA: not available. MA: microarray; WB: western blot; FA: functional assay including GSH assay, cAMP assay, Rho activation assay, adhesion assay and migration assay. Donors in bold were used in microarray experiment.
