# Supplementary material for: Susceptibility to glaucoma: differential comparison of the astrocyte transcriptome from glaucomatous African American and Caucasian American donors
Source: Genome Biol. 2008 Jul 9;9(7):R111. doi: 10.1186/gb-2008-9-7-r111 (PMC2530868; doi:10.1186/gb-2008-9-7-r111)
Supplement: Additional data file 3 — Probe-sets on the chip and used in analysis. [file gb-2008-9-7-r111-S3.doc]

***Table S3: Number of probe-sets on the chip and used in the analysis***

| Chip Name | Total number of probe-sets in the chip | Number of probe-sets used in the analysis | Number of genes used in the analysis* |
| --- | --- | --- | --- |
| Affymetrix Hgu133a2 | 22277 | 16688 | 10519 |
| Affymetrix Hgu95av2 | 12625 | 6629 | 5388 |

* Here the number of genes represents the number of unique Entrez IDs corresponding to the probe-set IDs.
