# Supplementary figures and images for: Susceptibility to glaucoma: differential comparison of the astrocyte transcriptome from glaucomatous African American and Caucasian American donors
Source: Genome Biol. 2008 Jul 9;9(7):R111. doi: 10.1186/gb-2008-9-7-r111 (PMC2530868; doi:10.1186/gb-2008-9-7-r111)

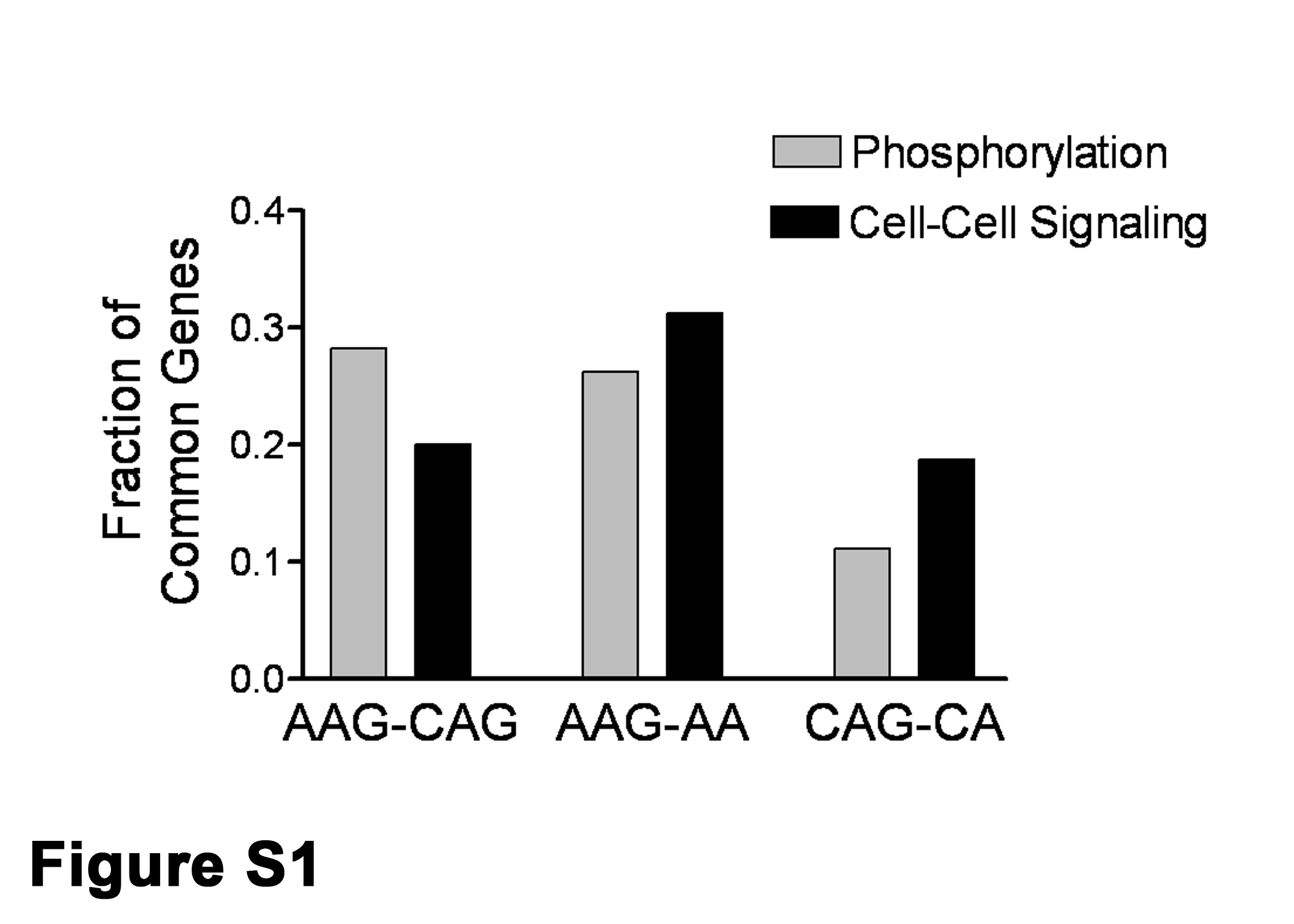

Supplement: Additional data file 10 — Common genes were selected from the GO lists (Additional data files 7-9) for each dataset (AAG-CAG, AAG-AA, and CAG-CA comparisons). The fraction of common genes (y-axis) for the GO terms 'phosphorylation' (grey bar) and 'cell-cell signaling' categories (black bar) are shown. [file gb-2008-9-7-r111-S10.tiff]

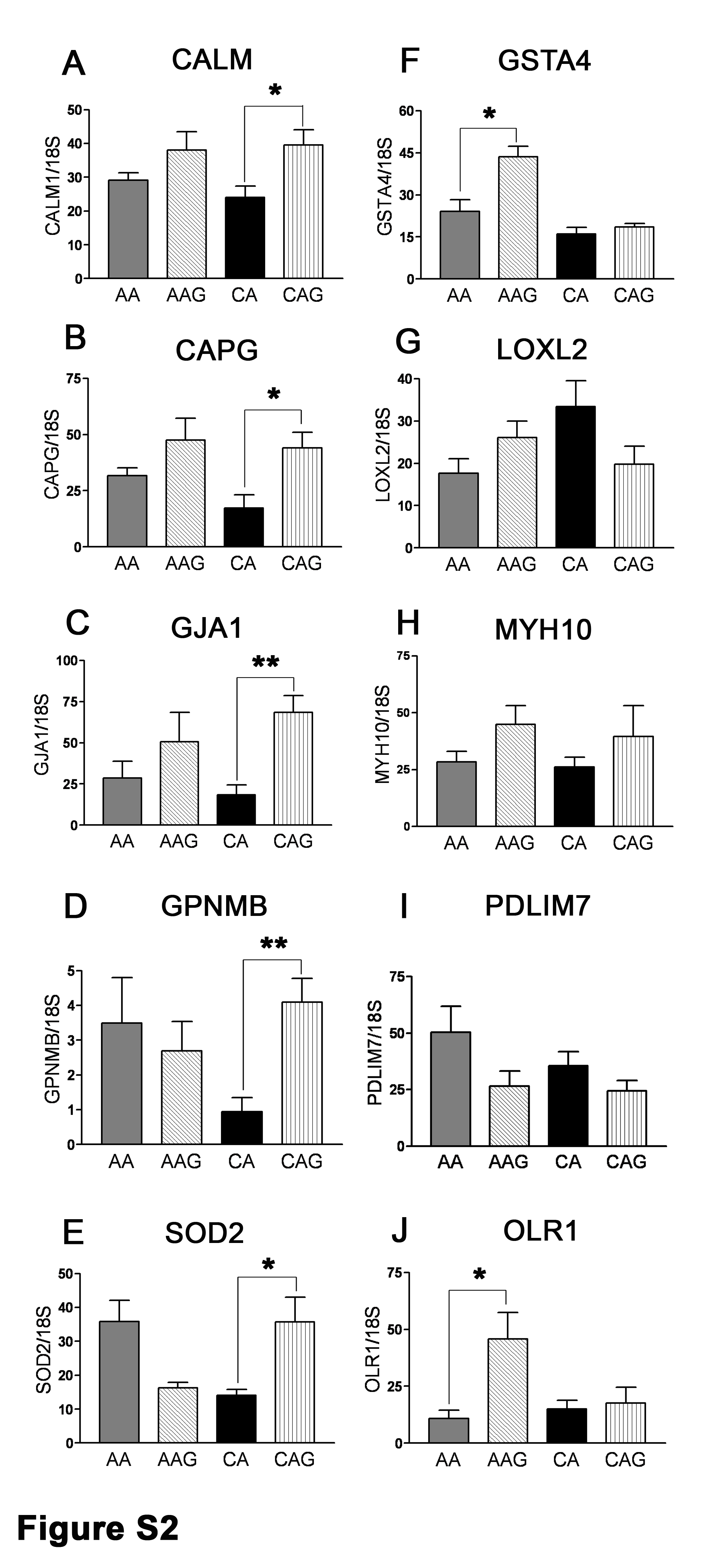

Supplement: Additional data file 11 — (a-e) CAG-CA and comparison: CALM (a), CAPG (b), GJA1 (c), GPNMB (d) and SOD2 (e). (f-j) AAG-AA comparison: GSTA4 (f), LOXL2 (g), MYH10 (h), PDLIM7 (i) and OLR1 (j). Genes were normalized to 18S. Graphical representation of the relative mRNA levels in normal and glaucomatous AA and normal and glaucomatous CA astrocytes (n = 6, two-tailed t-test was used. Asterisk indicates p < 0.05). [file gb-2008-9-7-r111-S11.tiff]
